# Pathogenic Mutations in Cancer-Predisposing Genes: A Survey of 300 Patients with Whole-Genome Sequencing and Lifetime Electronic Health Records

**DOI:** 10.1371/journal.pone.0167847

**Published:** 2016-12-08

**Authors:** Karen Y. He, Yiqing Zhao, Elizabeth W. McPherson, Quan Li, Fan Xia, Chunhua Weng, Kai Wang, Max M. He

**Affiliations:** 1 Department of Epidemiology and Biostatistics, Case Western Reserve University, Cleveland, Ohio, United States of America; 2 Center for Human Genetics, Marshfield Clinic Research Foundation, Marshfield, Wisconsin, United States of America; 3 Zilkha Neurogenetic Institute, University of Southern California, Los Angeles, California, United States of America; 4 Deparment of Molecular and Human Genetics, Baylor College of Medicine, Houston, Texas, United States of America; 5 Department of Biomedical Informatics, Columbia University Medical Center, New York, New York, United States of America; 6 Institute for Genomic Medicine, Columbia University Medical Center, New York, New York, United States of America; 7 Biomedical Informatics Research Center, Marshfield Clinic Research Foundation, Marshfield, Wisconsin, United States of America; 8 Computation and Informatics in Biology and Medicine, University of Wisconsin-Madison, Madison, Wisconsin, United States of America; CNR, ITALY

## Abstract

**Background:**

It is unclear whether and how whole-genome sequencing (WGS) data can be used to implement genomic medicine. Our objective is to retrospectively evaluate whether WGS can facilitate improving prevention and care for patients with susceptibility to cancer syndromes.

**Methods and Findings:**

We analyzed genetic mutations in 60 autosomal dominant cancer-predisposition genes in 300 deceased patients with WGS data and nearly complete long-term (over 30 years) medical records. To infer biological insights from massive amounts of WGS data and comprehensive clinical data in a short period of time, we developed an in-house analysis pipeline within the SeqHBase software framework to quickly identify pathogenic or likely pathogenic variants. The clinical data of the patients who carried pathogenic and/or likely pathogenic variants were further reviewed to assess their clinical conditions using their lifetime EHRs. Among the 300 participants, 5 (1.7%) carried pathogenic or likely pathogenic variants in 5 cancer-predisposing genes: one in *APC*, *BRCA1*, *BRCA2*, *NF1*, and *TP53* each. When assessing the clinical data, each of the 5 patients had one or more different types of cancers, fully consistent with their genetic profiles. Among these 5 patients, 2 died due to cancer while the others had multiple disorders later in their lifetimes; however, they may have benefited from early diagnosis and treatment for healthier lives, had the patients had genetic testing in their earlier lifetimes.

**Conclusions:**

We demonstrated a case study where the discovery of pathogenic or likely pathogenic germline mutations from population-wide WGS correlates with clinical outcome. The use of WGS may have clinical impacts to improve healthcare delivery.

## Introduction

Next-generation sequencing (NGS) technologies are increasingly used in biomedical research and clinical practice to identify disease-associated genetic variants for advancing precision medicine [1]. Precision medicine allows researchers and physicians to predict more accurately which treatment and prevention strategies for a particular disease will work in which groups of people based on their genetic differences [2]. More than 4,000 Mendelian disorders have been studied at the genetic level [3]. Assessment of genetic pathogenicity leveraging whole-genome sequencing (WGS), whole-exome sequencing (WES), or other sequencing data and electronic health records (EHRs) has recently become feasible as EHRs have been implemented widely in healthcare systems [4, 5].

Even though tens of millions of genetic variants are uncovered in the human genomes, we do not have a clear understanding of the majority of their roles in health and disease [6]. The American College of Medical Genetics and Genomics (ACMG) has recommended identification and return of incidental findings (IFs) in a set of 56 actionable genes [7, 8]. A study on the NHLBI Exome Sequencing Project (ESP) cohorts has reported actionable exomic IFs from 112 genes in 6,503 participants [9]. The spectrum of pathogenic genetic variations across a diverse set of genes spanning dominant and recessively inherited disorders in the Exome Aggregation Consortium (ExAC) population has been assessed [10]. A recent study that focuses on two genes suggests that up to 3% of individuals may be at risk for heart arrhythmias [5]. Germline mutations in 565 cancer-predisposition genes with an emphasis on the analysis of 60 autosomal dominant cancer-predisposing genes have been studied by Zhang et al. [11]. Other studies suggest that 1%-3% of the population may carry clinically actionable variants linked to Mendelian diseases [12–14]. Although these studies have provided significant insights into the human genome and have been able to identify individuals who carry clinically actionable genetic variants, their clinical impact remains unknown.

Our aims were to assess how often cancer gene screening identifies actionable cancer risk genes and to retrospectively evaluate whether the combination of WGS and EHR can facilitate improving prevention and care for patients with susceptibility to cancer syndromes. In this study, we firstly classified genetic pathogenicity of germline mutations in 60 autosomal dominant cancer-predisposition genes (S1 Table) among 300 deceased patients at Marshfield Clinic with WGS data using an in-house analysis pipeline called SeqHBase [15] based on the latest ACMG guidelines issued by the ACMG and the Association of for Molecular Pathology (AMP) [16]. Then we assessed clinical conditions for the patients who carried pathogenic or likely pathogenic variants using clinical data derived from their lifetime EHRs, followed by manual review of medical charts on selected patients by a MD clinical geneticist.

## Materials and Methods

### Sample Selection

In this study, 300 deceased patients were recruited from a Personalized Medicine Research Project (PMRP) [17–19] launched at Marshfield Clinic in 2002. The PMRP is a unique biorepository resource that includes serum, plasma, and DNA from over 20,000 patients with links to their EHRs, including diagnosis, treatment, procedure codes, laboratory values, prescriptions, pharmacy, and physician notes. This cohort represents a very stable population that are primarily non-Hispanic whites with over 70% claiming German ancestry [20]. Each of them has an average of over 30 years of dynamic, continuous, virtually comprehensive, and extractable EHR data as well as diet and activity data linked to participant biospecimens. Since their enrollment, about 2,000 PMRP participants have passed away, and over 1,000 of them have more than 30 years of longitudinal and nearly comprehensive EHR data. 300 of the 1,000 deceased patients were randomly selected, resulting in 161 females and 139 males. In addition, EHR is not being used to identify those patients. It could be biased towards including more patients with late-onset diseases (e.g. cancer) for collecting patients with having about 30-year EHRs. All participants in the PMRP had previously consented for research in written and this project was approved by Marshfield Clinic’s Institutional Review Board.

### Generation of 300 WGS Data

The blood samples of the 300 participants were sequenced by Complete Genomics (Mountain View, CA) according to manufacturer’s guidelines. The sequencing data were aligned to human reference (hg19). A minimum read-depth of 10 was used for variant calling. The VCF files, including single nucleotide variants (SNVs) and small insertions and/or deletions (INDELs), of the 300 genomes were provided by Complete Genomics. After quality control, over 27 million unique SNV and/or INDEL variants were identified across the 300 genomes.

### Computational Methods

NGS technology is an essential component supporting genomic medicine, but the volume and complexity of the data pose challenges for its use in biomedical research [21]. Sequencing a single human genome generates about 200 gigabytes of data. Therefore, enormous challenges for analyzing large-scale NGS and clinical data still exist including data storage, processing, scaling, quality control management, and interpretation [22]. It is critical to develop an efficient computational framework and tools to analyze large-scale sequencing and clinical data. To infer biological insights from massive amounts of NGS data and comprehensive clinical data in a short period of time, we developed an in-house analysis pipeline within a software framework called SeqHBase to quickly catalogue, retrieve and query genetic variants, and to help classify genetic pathogenicity based on the latest ACMG guidelines [16]. We used ANNOVAR [23] to annotate the 300 WGS data, then the variation and annotation information were managed and analyzed by the in-house system SeqHBase.

### Variant Classification

Variants in the 60 cancer-predisposition genes (S1 Table) were classified in the 300 genomes. In SeqHBase pipeline, there are a number of data quality filters, including minimum read-depth (e.g. reads > = 30X), maximum variant minor allele frequency (MAF; e.g., MAF < = 0.05%) in the 1000 Genomes Project [13], the ESP [24], and the ExAC [25], variant classification by ClinVar [26], and biological functions interested (e.g., splicing, nonsynonymous, stop-gain, stop-loss, and frameshift). We collected genetic variants of reads > = 30X, including (i) any variant present in the ClinVar database and annotated in one of the biological functions interested and (ii) new variants absent in the ClinVar database and annotated in the biological functions interested with MAF < = 0.5%, in the 60 cancer-predisposing genes. Variants departing from Hardy-Weinberg equilibrium (exact test P ≤ 1E-6) [27] were further filtered. All variants collected can then be classified as “pathogenic,” “likely pathogenic,” “uncertain significance,” “likely benign,” and “benign” using a combination of automated assessment and manual review, by following the latest ACMG guidelines [16].

## Results

### Germline Mutations in the 60 Cancer-Predisposing Genes

**Fig 1:**
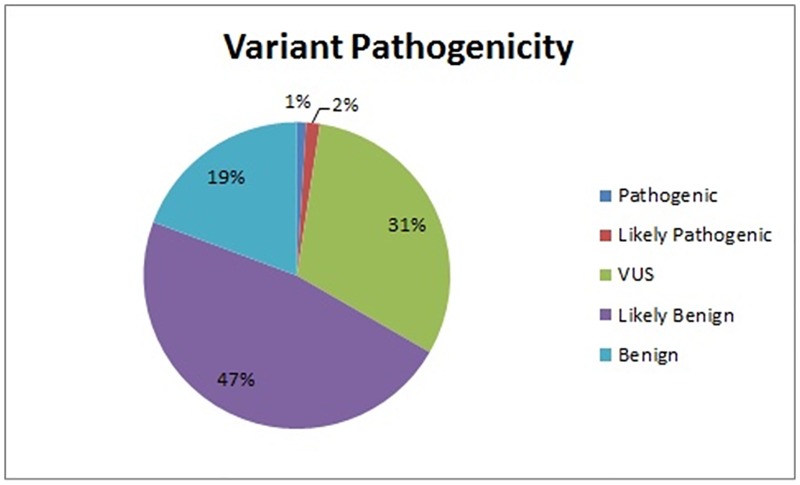
Pie chart of variant categories (pathogenicity) in 60 autosomal dominant cancer-predisposing genes. Provided are the percent of total variants for each category shown in S2 Table.

In the 300 whole genomes, the data presented in those biological functions interested across the 60 cancer-predisposing genes spanning diverse autosomal dominant cancers encompass 207 classified variants (S2 Table). Of the 207 variants in the 60 genes, 5 variants were classified as “pathogenic” or “likely pathogenic” (Fig 1). The 5 variants are shown in Table 1, and all of them are absent in the 1000 Genomes Project, the ESP, and the ExAC cohorts. To further confirm that our automated analysis is reliable, a certified medical geneticist manually reviewed the variant data and provided clinical interpretation on these variants (Table 2).

**Table 1:** Pathogenic/likely pathogenic variants in the 60 cancer-predisposing genes identified by WGS of 300 deceased patients*.

Gene	Chr	Position	AA Change	rs #	Function	Homs/Hets
*APC*	5	112102976	c.311C>G	rs74953290	p.Ser104Ter	0/1
*BRCA1*	17	41246018	c.1530del	rs80357735	p.Gly511Alafs	0/1
*BRCA2*	13	32913729	c.5238dupT	rs80359499	p.Ser1746Alafs	0/1
*NF1*	17	29679366	c.7486C>T	rs866445127	p.Arg2496Ter	0/1
*TP53*	17	7578389	c.541C>T	rs587782596	p.Arg181Cys	0/1

* Chr denotes chromosome, AA denotes amino acid, and rs # denotes rs number.

**Table 2:** Clinically Significant Variants Associated with Cancers.

Gene	Function	Observed Phenotypes (age-onset)	Manual interpretation by a certified medical geneticist
*APC*	p.Ser104Ter	Prostate cancer (early-70s); many colon polyps, developed colorectal cancer (mid-60s); family history of colon cancer (mid-50s)	This variant is located within the exon 3 of the *APC* gene, and will cause a truncated gene product. This variant has been found in a patient with familial adenomatous polyposis (FAP), PMID 21142386. Truncating variants in *APC* are the cause of FAP and are high risk factor for colorectal cancer (CRC), which is consistent with this individual's phenotype and family history. In addition, other truncating variants in exon 3 of *APC* have been found in patients with FAP/CRC (PMID 9664575, 25559809, 21646762 and 20649969). Therefore, this variant is interpreted as pathogenic.
*BRCA1*	p.Gly511Alafs	Breast and ovarian cancers (early-50s)	This variant is located within the exon 11b of the *BRCA1* gene, and will cause a truncated gene product. This variant has been found in a patient from a cohort of breast/ovarian cancers, PMID 12698193. Truncating variants in *BRCA1* are high risk factor for breast/ovarian cancers, which is consistent with this individual's phenotype. In addition, other truncating variants in exon 11b of *BRCA1* have been found in patients with breast/ovarian cancers (PMID 18489799, 15117986, and 8880569). Therefore, this variant is interpreted as pathogenic.
*BRCA2*	p.Ser1746Alafs	Prostate cancer (early-70s)	This variant will cause a truncated gene product of *BRCA2*. This variant has been found in multiple patients with breast/ovarian cancers, PMID 11802209, 23110154 and 24504028. Truncating variants in *BRCA2* are high risk factor for breast/ovarian cancers and prostate cancer, which is consistent with this individual's phenotype. Therefore, this variant is interpreted as pathogenic.
*NF1*	p.Arg2496Ter	Multiple skin (both basal cell and squamous) cancers (mid-70s)	This variant is located within the exon 51 of the *NF1* gene, and will cause a truncated gene product. This variant has been found in at least two patients with neurofibromatosis 1, PMID 7981679 and 22965773. Truncating variants in *NF1* cause neurofibromatosis 1, and are high risk factor for skin cancers, which is consistent with this individual's phenotype. In addition, other truncating variants in exon 51 of *NF1* have been found in patients with neurofibromatosis 1 (PMID 23656349 and 7981679). Therefore, this variant is interpreted as pathogenic.
*TP53*	p.Arg181Cys	Pancreatic cancer (mid-80s and mid-80s), the patient’s child died from melanoma in 30’s	This variant will cause a missense change from Arg to Cys at the codon 181 of the *TP53* gene product. This variant has been found in the germline of patients with different types of cancers from multiple unrelated families (PMID 7981679, 27157322, 27501770, 23484829 and 22965773). This variant was not found control databases including ExAC and 1000 genome. Defects in *TP53* cause Li-Fraumeni Syndrome, and are high risk factor for multiple types of cancers including pancreatic cancer and melanoma, which is consistent with this individual's phenotype and family history. In addition, studies suggest that the Arg181Cys change caused deficiency of *TP53* function (PMID 10229196, 12909720 and 21343334). Therefore, this variant is interpreted as pathogenic.

### Clinical Impact

5 of the 300 participants carried presumed “pathogenic” or “likely pathogenic” variants in the 60 autosomal dominant cancer-predisposing genes. The 5 “pathogenic” or “likely pathogenic” variants identified in this study are located in 5 genes including *APC*, *BRCA1*, *BRCA2*, *NF1*, and *TP53*. The *APC* gene encodes a multi-domain protein that plays an essential role in tumor suppression by antagonizing the WNT signaling pathway [28]. Inappropriate activation of this pathway through loss of *APC* function contributes to cancer progression, as in familial adenomatous polyposis [29]. Mutations in *APC* may result in colorectal cancer [30], prostate cancer [31], and other cancers [32]. The *BRCA1* gene is a protein product and is responsible for DNA repair [33]. It forms several distinct complexes through association with different adaptor proteins, and each complex forms in a mutually exclusive manner [34]. Mutations in *BRCA1* may result in breast and/or ovarian cancer [35] and pancreatic cancer [36]. The *BRCA2* gene is also a protein product responsible for DNA repair [37]. It is a key mediator of homologous recombination [38]. Mutations in *BRCA2* may result in breast and/or ovarian cancer [39], pancreatic cancer [40, 41], and prostate cancer [42] as well. The *NF1* gene encodes neurofibromin, a cytoplasmic protein that is predominantly expressed in neurons, Schwann cells, oligodendrocytes, and leukocytes. Mutations in *NF1* may result in juvenile myelomonocytic leukemia [43], neurofibromatosis [44], Neurofibromatosis-Noonan syndrome [45], and Watson syndrome [46]. Interestingly, a multidisciplinary team at Yale University, led by Yale Cancer Center members, has confirmed that *NF1* is a “major player” in the development of skin cancer [47], which is also observed in this study. The *TP53* gene responds to diverse cellular stress to regulate target genes that induce cell cycle arrest, apoptosis, senescence, DNA repair, and/or changes in metabolism [48]. Mutations in *TP53* may result in adrenal cortical carcinoma [49], breast cancer [50], choroid plexus papilloma [51], colorectal cancer [52], hepatocellular carcinoma [53], Li-Fraumeni syndrome [54], nasopharyngeal carcinoma [55], osteosarcoma [56], pancreatic cancer [57], basal cell carcinoma [58], and glioma susceptibility [59].

The 5 patients, who carried “pathogenic” or “likely pathogenic” variants, were expected to express autosomal dominant cancer-predisposing syndromes based on their genetic profiles. We reviewed the 5 patients’ lifetime EHRs and found the following results: (i) One male patient who carried a stop-gain mutation (rs72953290) in *APC* had prostate cancer, many colon polyps, and colorectal cancer. In addition, he had family history of colon cancer. (ii) One female carried a frameshift deletion (rs80357735) in *BRCA1* that is predicted to result in a significantly increased risk for breast and ovarian cancer. In fact, this patient had no known family history of breast cancer and did not receive yearly breast exams or mammograms. She was diagnosed with breast and ovarian cancer in her early-50s and died 6 years later. (iii) One male patient, who carried a frameshift insertion (rs80359499) in *BRCA2*, had prostate cancer in his early-70’s and died a few years later. (iv) One male patient, who carried a stop-gain mutation (rs866445127) in *NF1*, had multiple skin (both basal cell and squamous) cancers. (v) One female patient, who carried a missense mutation (rs587782596) in *TP53*, had pancreatic cancer. Additionally her child died from melanoma in his 30’s. The summary information is presented in Table 2. Namely, each of the 5 patients had one or more different types of cancers, demonstrating consistency with their genetic profiles.

Even though there are no family members in the 300 deceased patients, it is conceivable that family history recorded in EHRs can help disease prevention. As mentioned in the 2^th^ patient who carried the cancer-predisposing mutation in *BRCA1*, she died from breast and ovarian cancer in her later 50’s. This case implied that it could be important to have aggressive screening and prophylactic surgery for patients with *BRCA1* mutations. In addition, it is very likely that more aggressive surveillance or preventative measures could have extended the lives of those patients if genetic testing had been done in their earlier ages. Therefore, combining WGS and EHRs could potentially improve personalized healthcare.

## Discussion

Combining the functional characterization of identified genetic variants with comprehensive clinical data available in EHRs has the potential to provide compelling evidence to implicate novel disease-associated variants in phenotypically well-characterized patients. In this study, we analyzed germline mutations in the 60 autosomal dominant cancer-predisposition genes in 300 deceased patients with WGS data and nearly complete long-term medical records. To infer biological insights from massive amounts of WGS data and comprehensive clinical data in a short period of time, we developed an in-house analysis pipeline within a software framework called SeqHBase to quickly classify genetic pathogenicity based on the latest ACMG guidelines [16]. The pathogenic and/or likely pathogenic variants identified in this study were further reviewed using the carriers’ lifetime EHRs. Of the 300 participants, each of the 5 (1.7%) carried a presumed “pathogenic” or “likely pathogenic” variant in one of the 60 cancer-predisposing genes. When assessing extensive clinical data, each of the 5 patients had one or more different cancers, exhibiting fully consistency with their genetic profiles. The results generated in this study demonstrated that genetic mutations in autosomal dominant cancer-predisposing genes could be potentially used in clinical diagnosis, prevention, and personalized treatments, showcasing the power of combining WGS and EHR to accelerate biomedical discoveries. It also showed potential impacts of clinically actionable genetic variants over a lifetime and demonstrated that genomic sequencing could be helpful in precise disease diagnoses and risk prediction. Meanwhile, we have realized that the 300 patients selected in this study should not be regarded as a representative population at Marshfield Clinic. That is, younger people may need to be recruited with WGS study in the future.

We acknowledge that WES or targeted sequencing may provide similar results at a reduce cost. However, WES or targeted sequencing technologies may not capture the whole genome coding regions comprehensively while WGS generates more complete coverage for the whole genome regions [60]. Although sequencing costs have dropped substantially in the past a few years, the cost for data analysis and interpretation remain very high. Further comprehensive studies are needed as true impacts on clinical outcomes may be much more complex. It may not be feasible to use WGS for screening general population in clinical practice now.

In addition, more efforts are needed to distinguish genetic variants that are truly clinically actionable, that is, the variants are useful for guiding clinical decisions regarding interventions to improve health outcomes. As multiple independent evaluations might have classified variant pathogenicity differently [5], more stringent criteria and the latest ACMG guidelines should be compiled prior to reporting pathogenic variants [61].

In summary, clinical research studies of the implementation of genomic data in healthcare can provide valuable lessons on how genomic data should be managed, and patient privacy should be protected, when incorporating genomic data into clinical practice on a larger scale. These lessons can alert healthcare institutions of the scientific and technical challenges of using genomic data in precision medicine. NGS technological advances in clinical genome sequencing and adoption of EHRs will pave the way to create patient-centered precision medicine in clinical practice. The rise of *Big Data* in NGS and clinical data will contribute to better treatment paradigms, leading to improvements in diagnosis and personalized treatments that may ultimately lead to an overall reduction in healthcare cost. This study portrayed a promising method for assessing genetic pathogenicity by using WGS data.

## Supporting Information

S1 Table 60 Autosomal Dominant Cancer-Predisposition Genes. (XLS)

S2 Table Genetic Variants of the 300 Patients Across the 60 Cancer-Predisposition Genes. (XLS)

## Abbreviations

ACMG: American College of Medical Genetics and Genomics
EHRs: electronic health records
NGS: next-generation sequencing
WES: whole-exome sequencing
WGS: whole-genome sequencing
